# Vibronic Effects Analysis of the Substituent Effect on the Spectral Properties of the EMI and CPL of Three [7]Helicene Derivatives

**DOI:** 10.3390/molecules30010044

**Published:** 2024-12-26

**Authors:** Qiushuang Xu, Meishan Wang, Yanli Liu

**Affiliations:** 1 Department of Physics, Yantai University, Yantai 264005, China; 2 College of Integrated Circuits, Ludong University, Yantai 264005, China; 3 School of Physics and Optoelectronics Engineering, Ludong University, Yantai 264005, China; yanliliu@ldu.edu.cn

**Keywords:** vibronic effect, substituent effect, adiabatic Hessian, circularly polarized luminescence

## Abstract

The substituent effect has a significant influence on the optical properties of spectral shape, width, and wavelength, and the intensities of the maximum peaks of emission (EMI) and circularly polarized luminescence (CPL). In this work, we conducted a systematic theoretical study to investigate how substituents alter the optical response in the EMI and CPL spectra of three [7]helicene derivatives at the vibronic level. To incorporate the vibronic effect, a state-of-the-art time-dependent (TD) method was used to achieve the fully converged spectra. In the meantime, a time-independent (TI) approach also provided a way to show the progression of the spectra, serving as a complementary strategy and creating reliable documentation for the experiment. The experimental spectra of EMI and CPL are nicely reproduced, which validates the reliability of the Adiabatic Hessian (AH) model in simulating experimental data. This allowed us to analyze in detail the effect of substituents, particularly on the optical responses. The introduction of cyano and methoxy groups is highlighted, as they altered the transition dipole moments and led to a 1000-fold increase in the intensity of EMI and CPL. Moreover, substituents can also rationally alter the spectral shape of EMI and CPL by affecting the responsible normal modes. The unique CN stretching and the MeO rotation in the substituted [7]helicene are highlighted as key factors contributing to the different behaviors of EMI and CPL. This sheds light on the design and synthesis of helicenes that can serve as ideal full-color EMI and CPL emitters.

## 1. Introduction

The substituent effect is one of the most important and widely used terms for rational control of the photophysical properties of materials in organic chemistry [1]. For instance, the cyano [2] and methoxy groups [3] exhibit electron-withdrawing characteristics and donating properties, respectively. They are capable of significantly modifying electronic structures and optical properties, particularly for the fabrication of highly efficient circularly polarized luminescence (CPL)-active materials [4,5]. Helicenes are a typical type of preferred CPL material for the production of CPL. This is due to their excellent chiroptical response. They are ortho-fused polycyclic aromatic compounds and exhibit prominent chiroptical properties because of their high rotational strength from the inherent electronic transitions along the helical π-conjugated skeleton. They have a wide variety of applications in different kinds of areas, such as chiral molecular recognition [6,7], molecular machines [8,9], organic light-emitting diodes (OLEDs) [10,11], and so forth. Recently, their chiroptical and optical properties have been increasingly highlighted in the current literature, such as in molecular sensors and switches. However, unsubstituted helicenes are reported to suffer from low-fluorescence quantum yield (QY) and the dissymmetry factor, which severely hinders the application of helicenes and their derivatives. Fortunately, the introduction of electron-withdrawing and electron-donating substituents into the helical framework is an effective way to improve QY and unity along with the tunable dissymmetry factor for CPL [12]. For example, the QY values of unsubstituted [5]helicene, [6]helicene, and [7]helicene are only 0.04, 0.04, and 0.02, respectively [13,14,15]. When substituents are introduced, the values of QY and the CPL properties are greatly improved. For example, by introducing electron-donating groups and electron-withdrawing groups at appropriate substitution positions of [7]helicene, the QY value can be increased from 0.02 to 0.17 [15].

It is well known that theoretical investigations have been successfully used to explain the origin of the chiroptical responses of chiral materials [16,17,18]. For instance, the Herzberg–Teller effect on the spectral shape of different azahelicenes [19], the symmetry of double helicenes [20], and the solvent effect on the wavelength of the maximum of EMI/CPL emission [21,22,23] emphasize the importance of the vibronic effect in analyzing the spectral properties of helicenes. However, the effect of cyano and methoxy group substituents and their derivatives on [7]helicene remains elusive. Intriguingly, the experimental work performed by Matsuda et al. [15] has shown us that the addition of a methoxy group and/or a cyano group can endow unsubstituted [7]helicene with enhanced CPL properties. Therefore, it is of great interest to gain further insight into the substituent effect of cyano and methoxy groups on the optical properties of [7]helicene at a vibronic level. This is very helpful in rationally and effectively designing and synthesizing CPL-active helicenes.

**Figure 1:**
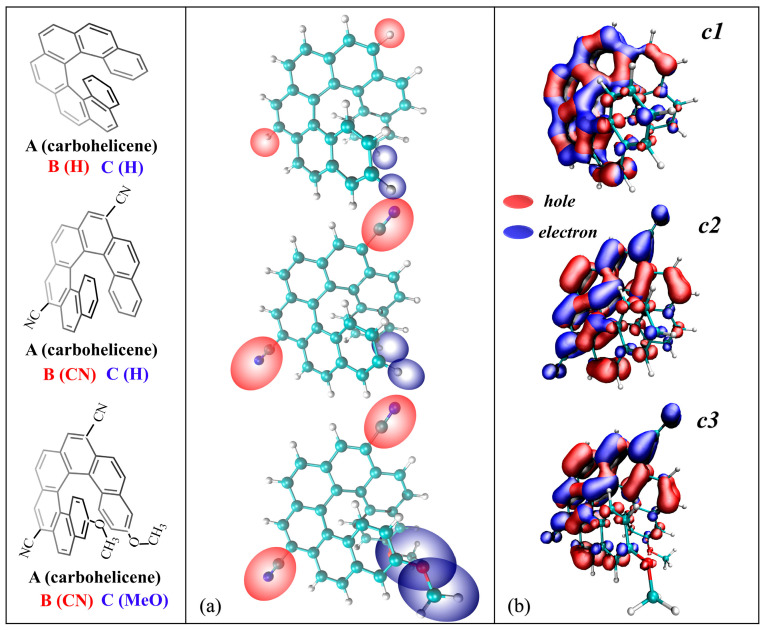
(**a**) Artificially divide the three [7]helicene derivatives into three fragments. (**b**) Hole and electron distributions (isovalue = 0.001 a.u.) from the relaxed S_1_ to the GS under S_1_-minima for the three [7]helicene derivatives. The red area represents the “hole” and the blue area represents the “electron”.

In this work, we aim to report a thorough computational study of vibronic coupling and substituent effects in modifying the emission (EMI) and CPL spectral properties of three [7]helicene derivatives and to provide valuable insights through phenomenological considerations related to their spectral shape, width, position, intensity of maximum peaks, and responsible modes. The helicenes of interest are unsubstituted [7]helicene (***c1***), 7,12-dicyano [7]helicene (***c2***), and 7,12-dicyano-3,16-dimethoxy [7]helicene (***c3***), as shown in the left panel of Figure 1. The time-independent (TI) and time-dependent (TD) approaches are two effective methods for showing the progression of the spectra, which are used to study the vibrationally resolved EMI and CPL spectra. In the TI approach [24,25,26,27], the spectrum is modeled as the cumulative outcome of individual vibronic transitions, each of which contributes to the overall spectral profile through their superposition. This method has been successfully used to identify the contributed vibrational modes of organic molecules and dyes [28,29,30]. Notably, it should be noted that the convergence problem might happen with the TI method, especially for large systems such as helicenes. In contrast, the TD approach [31] can be used to obtain fully converged theoretical spectra with the Fourier transform of the transition dipole correlation function. However, the TD strategy alone is not able to make a direct assignment to the spectrum. The combination of these two approaches, TI and TD, allows us to fully understand the vibronic effects in the interpretation of the experimental chiroptical responses. The Franck–Condon (FC), Herzberg–Teller (HT) [32], and Duschinsky effects [33] are all accounted for during the investigation into the vibrationally resolved spectra of EMI and CPL. To further reveal the paramount impact of the FC and Duschinsky effects on the spectral properties, the state-of-the-art computation of the adiabatic Hessian (AH) model [19,34], adiabatic shift and frequency (ASF), and adiabatic shift (AS) models [35] is employed for the present case.

## 2. Results and Discussion

### 2.1. Transition Nature

To shed light on the effect of cyano and methoxy groups on the fundamental nature of the transition, an inter-fragment charge transfer (IFCT) [36,37] method based on the Multiwfn program [38,39] was employed. IFCT revealed that the electrons on fragment A (carbohelicene core) experience a significant redistribution with a value larger than 0.9 for all three derivatives (Table S1). This suggests that the electronic structures of the carbohelicene core are significantly adjusted during the emission, which means the carbohelicene core plays a vitally important role in the process of emission. One can see that when two cyano groups are introduced to ***c1***, the electrons are transferred from fragment A (carbohelicene core) to B (cyano group) during emission. Moreover, the transferred electrons of ***c2*** from fragment A to B increase from 0.00039 to 0.069 after being substituted by cyanos with respect to ***c1***. This supports the electron-withdrawing effect of the cyano groups. When methoxy groups are further introduced to ***c2***, 0.066 electrons are transferred from cyano groups to the carbohelicene core, and 0.00377 electrons are transferred from the carbohelicne core to methoxy (***c3*** in Table S1). The difference in charge transfer between ***c2*** and ***c3*** is mainly due to the electron-donating effect of the methoxy groups. The results are consistent with those of the transition nature of ground state (see Table S4 and Figure S4 for details).

For a better visualization, the distribution of holes and electrons is transformed into a smooth Gaussian function distribution in Figure 1. The red area represents the “hole” where the electron leaves while the blue area represents the “electron” where the electron goes. The distribution of the holes and electrons of ***c1*** is plotted as a reference molecule. When cyano groups are introduced to ***c1***, one can see from Figure 1b for ***c2*** that only a blue isosurface appears in the fragment B supporting the electron-withdrawing effect of the cyano groups. This is consistent with the results of IFCT. For fragment A, both blue and red isosurfaces spread over its whole helical skeleton. This indicates that the carbohelicene core accepts some electrons from the cyano groups and also transfers some electrons to the cyano groups, which leads to a significant redistribution of the carbohelicene core. It is noteworthy that the red isosurfaces are larger than the blue, which indicates the loss of electrons from the carbohelicene core during the excitation to ground state. These results are in line with the data reported by IFCT in Table S1. For ***c3***, fragment A is blue and fragment C is red, supporting the electron-withdrawing and electron-donating effects of these two substituents, respectively.

### 2.2. Calculated Spectra

**Figure 2:**
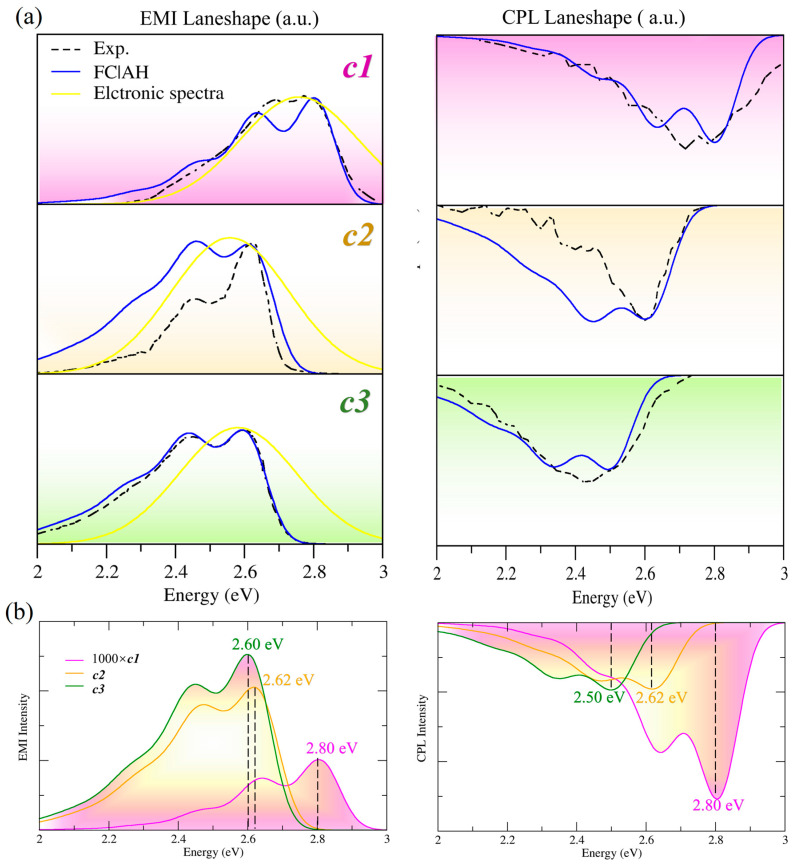
(**a**) The vibrationally resolved EMI and CPL spectra, obtained with the FC|AH model, in chloroform, together with the experimental spectra (Exp.) and the pure electronic emission spectra convoluted with a Gaussian with the HWHM of 0.20 eV (labeled as TD-DFT). (**b**) Comparison of the theoretical EMI and CPL intensities of ***c1***, ***c2***, and ***c3***.

The calculated pure electronic spectra and vibrationally resolved spectra are compared to the experimental ones in Figure 2. It is acknowledged that the current theoretical framework, albeit insightful, is unable to precisely replicate the exact positional information obtained from the experimental findings. Therefore, the pure electronic and vibronic spectra of EMI and CPL have also been shifted for a better visualization with the experiment. The theoretical spectra have been redshifted by 0.44 eV for ***c1***, 0.35 eV for ***c2***, and 0.37 eV (EMI) and 0.47 eV (CPL) for ***c3***. The pure electronic emission spectra of the three molecules are contributed by the transitions from the low-lying excited states (S_1_→S_0_). The electronic excitations of S_1_→S_0_ are dominated by the highest occupied molecular orbital (HOMO)→the lowest unoccupied molecular orbital transitions (LUMO) transitions, mainly have (π, π*) and intramolecular charge transfer (CT) characteristics as Figure S5 in SM (Supplementary Materials) shows. One can see from the top panel of Figure 2 that the pure electronic spectra of the three molecules are all predicted with only one emission peak. This indicates that the pure electronic calculation alone cannot reproduce the rich patterns observed in the experiment. This is to be expected since the vibronic coupling has been shown to play a crucial role in the simulation of the fine structures reported by experiments [40,41,42,43]. When the vibronic effect is taken into consideration, the agreement with the experiment is significantly improved. The spectral shape, spectral bandwidth, and even the number and the relative position of main peaks are all in better agreement with the experimental findings for all cases. As one can see from Figure 2, FC alone is able to reproduce the experimental spectra well and capture the vibrational structures. Therefore, our discussion will mainly focus on the vibrationally resolved EMI and CPL spectra, obtained with the FC|AH model.

Let us first discuss the performance of the computed EMI and CPL spectra of molecule ***c1***. We note that the theoretical spectra of ***c1*** give a good simulation to the experimental ones. For instance, both the spectral width and the number of the emission peaks have been nicely captured for EMI. The downside is that the experimental spectrum of EMI shows two relatively high geometrically consistent peaks at around 2.68~2.77 eV. In comparison, the theoretical EMI is predicted with a very high resolution at 2.63 eV and 2.80 eV, respectively. On the other hand, for the CPL spectrum, the theoretical CPL spectrum has nicely captured the spectral width and sign, albeit with a higher resolution than the experimental one. The results of our calculations for ***c1*** for both EMI and CPL are satisfying and in good agreement with the experimental ones.

For ***c2***, the theoretical result has captured the two experimental peaks at 2.62 eV and 2.45 eV for EMI well; however, it overestimates the height of the low-energy peak at 2.45 eV. In addition, the FC spectrum shows a slightly larger spectral width than the experimental EMI. Similarly, we also overestimated the spectral width of CPL. In order to find the reason for this overestimation, we then performed extra calculations by a further comparison with the ASF and AS models (shown in Figure S1). It can be seen that the results of the AS and ASF models are basically consistent, narrowing the spectral broadening and modifying the relative heights of the two experimental peaks. This highlighted that they highly improved the agreement with the experiment. The reasons for this phenomenon can be explained by the nature of the different models. These depend on the relative effects of the Duschinsky transformation and frequency changes and displacement between GS and ES. In specific terms, AH comprehensively considers the effects of frequency changes and displacement between GS and ES, as well as the Duschinsky effect. While ASF ignores the Duschinsky transformation by setting the Duschinsky matrix to 1, the AS model only accounts for the displacement between GS and ES. The comparison results show that the AS and ASF models are in good agreement with the experiments due to their own characteristics. Furthermore, we conducted additional tests to examine the impact of the Vertical model on the spectral shape, with the Vertical model avoiding the cumbersome optimization of ES geometry compared to the Adiabatic model. Vertical Hessian (VH) and Vertical Gradient (VG) are adopted. Both VH and VG assume that the initial and final states have the same harmonic PES curvatures, while, in the same way as AH, VH considers the effects of frequency, displacement, as well as the Duschinsky, but VG only considers the small displacements along the directions of the normal coordinates. As shown in Figure S2, the VG model yields almost identical results to the AS model. Similarly, the VH and AH models produce comparable outcomes, both of which overestimate the spectral width. Specifically, these results further confirm our previously discussed findings. In addition, the HT effect can adjust the relative height of the spectra, thereby improving the agreement with the experiment, especially for weak transitions. However, the comparison of the emission spectra of the system studied in this paper, FCHT (FC and HT), shows that for the present strong-state system, the HT effect is negligible (Figure S3).

Among the three molecules, the performance of FC|AH for ***c3*** is the best. Whether it is the spectral broadening, spectral shape, spectral sign, the rich fine structures, or the relative height of the experimental two peaks in the EMI spectrum, the theoretical EMI of ***c3*** is highly consistent with the experiment. However, the theoretical result for CPL exhibits two peaks with a signal negative sign, whereas the experimental CPL is reported with only one primary peak and a pure negative sign at 2.43 eV. This discrepancy may be due to experimental noise affecting the CPL measurements.

The above results indicate that the FC|AH model is sufficient to provide a reliable and accurate description of the spectra for ***c1***, ***c2***, and ***c3***. This provides a solid theoretical foundation for further investigation of the effect of substituents on EMI and CPL.

### 2.3. Substituent Effect

The introduction of electron-donating and -withdrawing groups is reported to alter the optical properties of unsubstituted carbo [7]helicene. To fully understand the substituent effect on the behavior of EMI and CPL, we report the theoretical spectra of the three derivatives in terms of intensity in the bottom panel of Figure 2 so that the relative intensity can be captured. Note that, the spectra reported in terms of intensities have also been shifted to match the experimental spectra.

The calculated spectra in the bottom panel of Figure 2 (left panel) clearly show us that the introduction of the substituents largely enhances the intensities of the maximum peaks of EMI spectra. It should be noted that the intensity of ***c1*** is multiplied by a factor of 1000 to facilitate a better comparison with the two substituted [7]helicenes. As one can see, the intensity of the maximum peak of ***c2*** is greatly enhanced by three orders of magnitude with respect to that of ***c1***. Further introducing two methoxy groups to [7]helicene, the intensity of the maximum peak of the EMI spectrum continues to increase. This is consistent with the total FC intensities reported in Table 1, where ***c2*** and ***c3*** are in the same order of magnitude and three orders of magnitude larger than ***c1***. This is due to the fact that the transition dipole moments *μ* (a.u.) are rationally altered by introducing cyano (***c2***) and methoxy groups (***c3***) into unsubstituted carbo [7]helicene (***c1***) (Table 1). This suggests that the introduction of electron-donating and electron-withdrawing groups at the appropriate substitution positions, which alters the nature of the S_1_→S_0_ transition for [7]helicene, is a promising way to alter the optical properties of unsubstituted carbo [7]helicene.

It is interesting to note that the wavelengths of the maximum EMI peaks show a relative shift among the three [7]helicene derivatives. The introduction of cyano groups leads to a redshift of ***c2*** to ***c1***, which is in good agreement with the experiment. A further introduction of methoxy groups only leads to a rather small shift between ***c2*** and ***c3***. This can be understood with the help of the adiabatic energies (*E_AH_*) reported in Table 1. For instance, the smaller *E_AH_* of ***c2*** with respect to ***c3*** is the main reason responsible for the small blueshift of ***c3*** with respect to ***c2***.

Now let us focus on the changes in the spectral shape. It can clearly be seen from the experiment that when only two CN substituents are introduced (in ***c2***), the height of the shoulder peak at a low energy of EMI is significantly reduced. This has been nicely captured by the FC|AS model for ***c2***. After the further introduction of two methoxy groups, the relative intensities of the two peaks in the EMI spectrum are altered, resulting in a similar spectral shape to that of ***c1***. The effect on the spectral shape is qualitatively captured by the theoretical results. A more detailed explanation of the reason for the substituent effect can be ascribed to the assignment of the main vibronic bands in Section 2.4.

A similar discussion of substituents can be extended to CPL. The introduction of cyano and methoxy groups enhances the spectral intensities of CPL. The intensity of CPL for ***c1*** is much lower than that for ***c2*** and ***c3*** by a factor of 1000, with respect to the two substituted [7]helicenes. In the same way as EMI, AS provides a better performance in terms of CPL than the AH model, which is to be expected. Moreover, it is interesting to note that the spectral shapes of CPL are identical to those of EMI for all the systems considered here, except for the negative signs of CPL. What is more, similar spectral shifts are observed in the CPL spectra among these three [7]helicene derivatives, due to the different transition energies.

In addition, the zero-point energies (*E*_0→0_, eV) of the systems are also collected in Table 1. As one can see, the valves of *E*_0→0_ are altered by introducing the electron-donating and -withdrawing cyano and methoxy groups. This leads to the redshift of EMI and CPL for ***c2*** and ***c3*** with respect to ***c1*** from the vibronic level.

### 2.4. The Assignment of the Main Vibronic Bands

**Figure 3:**
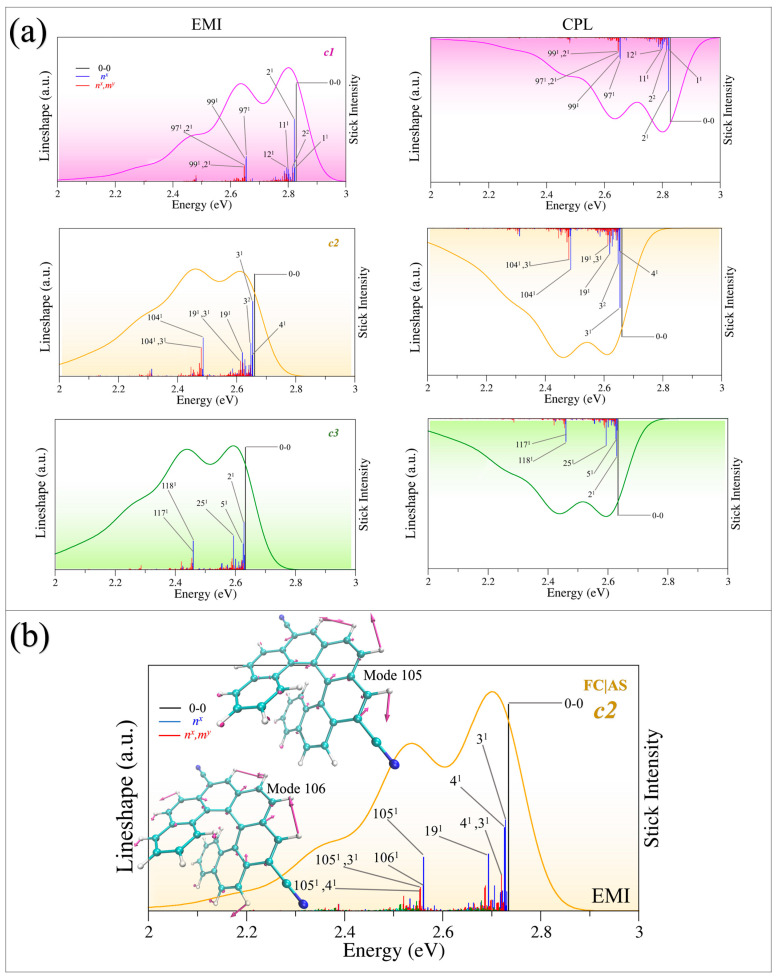
(**a**) Assignments of the main stick bands of EMI and CPL in FC|AH, calculated in chloroform. (**b**) Assignments of the main stick bands of EMI in FC|AS, calculated in chloroform.

A detailed analysis of the spectra can be made by utilizing the assignment of the main vibronic stick bands in Figure 3. Although the TD algorithm is more efficient than the TI algorithm in obtaining fully converged spectra, TI calculations can still be superior in identifying the different stick transitions and providing the main progressions along S_0_ modes for EMI and CPL. It should be noted that the TI method often encounters convergence problems; for instance, the convergence of ***c1*** calculated with FC|AH using the TI approach is only 62.3%, for ***c2*** it is 42.0%, and for ***c3*** it is 43.3%. Therefore, the above discussion of the substituent effect on spectral shape, position, and intensity in Section 2.3 was based on the TD method. The TI method was only used to sign the most contributed stick bands (Figure 3). Note that the intense stick bands in Figure 3 are reported as bars whose heights are proportional to the calculated intensity. The vibronic stick bands are labeled as “*n^x^*” and “*n^x^*, *m^y^*”; “*n*, *m*” represents the normal modes of S_0_ for emission spectra (EMI and CPL) and “*x*, *y*” are their number of quanta. *n^x^* represents the fundamental and overtone bands while *n^x^*, *m^y^* represent the combination bands.

**Figure 4:**
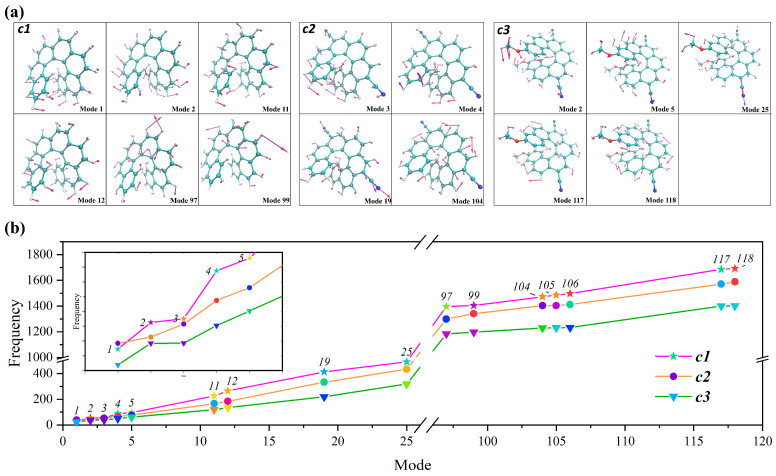
(**a**) Selected vibrational modes of ***c1***, ***c2***, and ***c3***. (**b**) The frequency change curve of selected vibrational modes relevant for the vibronic structures of the spectra in FC|AH.

We focus first on ***c1***, the most intense band of EMI is the 0→0 transition, followed by the transitions of <0|2^1^>, <0|97^1^>, and <0|99^1^>; the same happens to the CPL spectrum. Moreover, as can be seen from Figure 3a, the high-energy peak of the convoluted EMI spectrum at 2.8 eV can be attributed to the 0→0 transition and to progressions along the S_0_ low-frequency modes of 1 (34.55 cm^−1^), 2 (52.60 cm^−1^), 11 (226.42 cm^−1^), and 12 (264.79 cm^−1^). Mode 1 is the combination of CC and CH out-of-plane bending (wagging) of the terminal benzene rings (Figure 4). Modes 2, 11, and 13 are all combinations of CC and CH out-of-plane bending (wagging) of the whole helical skeleton. Furthermore, the low-energy shoulder peak at 2.65 eV mainly corresponds to the fundamentals of 97^1^, 99^1^, and the combination bands of 97^1^2^1^ and 99^1^2^1^. Modes 97 and 99 are both C-C stretching and C-C-H in-plane bending, as shown in Figure 4.

For the molecule of ***c2***, the high-energy peak of EMI located at 2.62eV is attributed to the transition of 0→0 and the fundamentals of the S_0_ modes of 3 (51.52 cm^−1^), 4 (67.47 cm^−1^), and 19 (333.37 cm^−1^), calculated with FC|AH. Figure 4 indicates that modes 3 and 4 are the combination of CC, CN, and CH out-of-plane bending (twisting). Mode 19 is attributed to the combination of CC and CN stretching and CH out-of-plane bending. The shoulder peak at 2.45 eV is mainly due to 104^1^ and the combination band of 104^1^3^1^. Mode 104 (1403.05 cm^−1^) corresponds to combinations of CC stretching and CH in-plane bending.

The main differences in the intense bands calculated with the AS and AH models are located at the contributions of the fundamentals of modes 4, 19, 104, 105 (1404.05 cm^−1^), and 106 (1412.37 cm^−1^). The intense vibronic bands of 4^1^ and 19^1^ are largely enhanced in the AS model, which modifies the relative intensities of the two peaks of EMI, improving agreement with the experiment (Table S3). Moreover, the contribution of modes 105 and 106 are also enhanced to improve agreement with the experiment in the AS model (Figure 3b). This indicates the enhancement of the contributed vibronic bands of 4^1^, 19^1^ and the reduced intensity of the high-frequency bands of 105^1^ and 106^1^ alters the relative height of the two peaks of EMI and leads to the better performance of the AS model with respect to the AH model.

The comparison between ***c1*** and ***c2*** shows us that the high-frequency energy band is lowered while the low-energy band of ***c2*** is enhanced with respect to that of ***c1***. The low-energy band of ***c1*** corresponds to the 0→0 transition and the progression modes of 1, 2, 11, 12, while that of ***c2*** corresponds to the modes 3, 4, 19, and the 0→0 transition. In the meantime, the high-frequency energy band in ***c1*** is mainly due to the fundamental modes of 97 and 99, while the responsible modes of ***c2*** are 105 and 106. As one can see, the frequencies of those responsible normal modes and the *E*_0→0_ of ***c2*** are lower than those of ***c1***, which suggests the redshift of ***c2*** to ***c1***. Moreover, the intensity of the 0→0 band is in line with the changes in dipole moments which were largely enhanced after substitution by cyano groups. Furthermore, the intensity of the 0→0 band and the relative displacements of main contributed modes in Table 1 and Table S3, indicate that the introduction of cyano groups alters the intensity of the contributed modes and leads to the change in the relative intensity of the two peaks in EMI. In addition, typical CN stretching and out-of-plane bending is observed in ***c2***, which is attributed to the cyano groups. This indicates that the reduction in the frequencies of the responsible modes and *E*_0→0_ leads to the redshift of ***c2*** against ***c1***. The enhancement of *I*_0→0_ and the intensity of contributed vibronic bands, as well as the CN stretching and out-of-plane bending are the main reasons for the difference in the EMI spectral shape and intensity of the different peaks of EMI between ***c2*** and ***c1***.

A similar discussion can be extended to molecule ***c3***; the high-energy peak is attributed to the 0→0 transition and the fundamental modes of 2, 5, and 25. Mode 2 is a combination of CC, CO, and CH bending and CN rotation. Mode 5 corresponds to CC, CO, CH out-of-plane bending, CN rotation, and a noteworthy rotation of methyl in the methoxy groups. For mode 25, CC, CN, CO stretching and CN rotation are observed. Moreover, the low-energy peak at 2.45 eV corresponds to 117^1^ and 118^1^. They are all combinations of CC stretching, CH in-plane bending, and CN rotations. The rotation of methyl in the methoxy groups is a typical and innovative movement of the responsible mode of molecule ***c3*** which is different from the other two molecules.

The main modes responsible for the EMI of ***c1***, ***c2***, and ***c3*** are different. In the same way as with ***c2***, the frequencies of the responsible vibrational modes are lowered in ***c3*** with the introduction of methoxy groups. This is due to the effect of the electron-withdrawing and electron-donating substituents, which modify the electron density and change the force constant, ultimately lowering the vibrational frequency (Figure 4 and Table S2). With the combined effects of these two substituents in molecule ***c3***, the red-shift of ***c3*** with respect to ***c2*** is very small. This is inconsistent with the *E*_0→0_ reported in Table 1. This demonstrates that the introduction of electron-donating and -withdrawing groups rationally alters the wavelength of the EMI maximum peak of unsubstituted carbo [7]helicene by altering the frequency of normal modes. Moreover, the intensity of 0→0 and the most intense vibronic bands are adjusted with the introduction of cyano groups, contributing to the different spectral shape of ***c3*** with respect to ***c1*** and ***c2***. Furthermore, the rotation of methyl in methoxy groups is another reason for the different spectral shape of ***c3***. Since EMI and CPL show similar spectral shapes under the FC (First-Order Cumulant) approximation, further elaboration on the specific attribution of CPL is avoided here.

## 3. Conclusions

In this work, we conducted a theoretical investigation into the effects of cyano and methoxy substituents on unsubstituted [7]helicene. The IFCT results indicate that the introduction of the withdrawing and donating groups promotes the electron transfer between the substituents and the carbohelicene core. This modifies the electron density of the helical skeleton of helicenes. The theoretical vibrationally resolved spectra show us that the substituents of cyano and methoxy groups have a great effect on the spectral shapes, intensities, and wavelengths of the maximum peaks for both EMI and CPL. The spectra reported in terms of intensity show us that the introduction of cyano and methoxy groups leads to a three orders of magnitude increase in dipole moment, which leads to the increase in the intensity of the maximum peaks of EMI and CPL from the electronic level. When the vibronic effect is accounted for, it becomes clear that substituents can alter the intensity of the 0→0 transition and the vibronic bands, thereby enhancing the overall intensities of the EMI and CPL spectra. This proposes a reliable strategy for designing strong CPL chromophores. Moreover, as one can see, the substituents also have an effect on the transition energy, *E*_0→0_, and the frequencies of the responsible modes, which leads to the redshift of the spectra of substituted [7]helicene of ***c2*** and ***c3*** with respect to ***c1***. This sheds light on the design of full-color EMI/CPL emitters. Furthermore, with the help of the assignment of the contributed modes, one can conclude that the CN stretching and out-of-plane bending, as well as the methoxy group rotation, are mainly responsible for the different behaviors of EMI and CPL. We believe that understanding the substituents from the vibronic level is useful for elucidating the influence mechanism of electron-withdrawing and -donating on the optical properties of helicenes, which can aid in the design of fluorescent materials with full-color EMI and CPL.

## 4. Computational Details

The electronic calculations of the three [7]helicene derivatives were conducted with Gaussian 16 software [44]. The minimum geometries of the ground state (GS) and excited state (ES) were optimized using density functional theory (DFT) and TD-DFT methods, respectively. Simultaneously, frequency calculations were performed to ensure that the true minima were obtained for both GS and ES. The solvation effect was taken into account using the linear-response polarizable continuum model (LR-PCM) [45], with calculations performed in chloroform, which is the same solvent used in the experiment [15]. In addition, LR-PCM was chosen because it comprehensively treats the effects of solvent on both the ground and excited states, is computationally efficient, and numerous studies [22,46,47] have shown that LR-PCM has proven accuracy in predicting electronic properties in solution. And to ensure uniformity, all optimizations and frequency calculations pertaining to GS and ES were conducted using the equilibrium solvent model. The CAM-B3LYP functional [48] in combination with the TZVP basis set [49] was applied during the calculations for the present work. The CAM-B3LYP/TZVP basis set has been applied throughout all the calculations, since it has been proven to make predictions with excellent results for vibronic calculations on helicenes [19,21,50].

The fully converged FC spectra were obtained by the TD approach while the information about the origins/contributions of the vibrational excitations was performed by the TI approach. The detailed computational methodology can be found in the Section 1 of SM. In order to understand and dissect the effect of Dusnchinsky, frequency changes, and displacement between GS and ES for the vibronic spectra, other than the AH model, the ASF and AS models were employed and compared in detail. ASF and AS are two simpler adiabatic models compared to AH.

It should be clarified that all the adiabatic models were developed to expand the initial and final-state PESs around their own equilibrium structures and are therefore expected to give accurate and reliable results. To be specific, the AH model accounts for the Duschinsky rotation, frequency changes between GS and ES, and the displacement between the two minima. With respect to AH, the AS model offers a closer approximation, assuming that the initial and final PESs have identical Hessians (meaning they share the same normal modes and frequencies) and their equilibrium geometries are merely shifted. Consequently, the vibrational structure of a spectrum derived from the AS model is predominantly influenced by the shifts in equilibrium geometries. The ASF model stands as a middle ground between the AH and AS models, taking into account variations in the harmonic frequencies of the two PESs but overlooking the Duschinsky mixing by assigning a value of 1 for the Duschinsky matrix. The comparison of the results calculated by these three models allows us to analyze and recognize the important factors in terms of interpreting the experimental data. In addition, the half-width at half-maximum (HWHM) of the convolving Gaussian was set to better match the experimental spectrum, with a value of 0.06 eV for ***c1***, ***c2***, and ***c3***. All the spectra calculations were performed by a version of the code ***FC****classes*3 [51]. All the vibronic spectra reported in this work refer to the M enantiomer.

To facilitate a precise and meaningful comparison between theoretical predictions and experimental spectral data, each vibronic spectrum is presented in a standardized format of normalized line shapes. The experimental data ($\phi_{\lambda }\left(\lambda \right)$) [52,53,54] were also transformed into the frequency domain. The transformation was performed with the formula $L_{EMI/CPL}\left(\omega \right)=N_{\chi }\omega^{-5}\phi_{\lambda }\left(\lambda \right)$. The $N_{\chi }$ in the equation is a normalization factor to ensure that the integral over the frequency domain is 1.

## Figures and Tables

**Table 1:** The electric dipole moments (*μ*, a.u), magnetic dipole moments (*m*, a.u), adiabatic energy (*E_AH_* eV), total FC intensity $I_{tot}^{FC}$, zero-point energy (*E*_0→0_, eV), and intensity of *E*_0→0_ ($Int_{E_{0\to 0}}$) from the lowest vibrational state of GS to the lowest vibrational state of ES.

Molecules	$\left|\mu \right|$	|*m*|	*E_AH_*	$I_{\mathrm{tot}}^{FC}$	***E*_0→0_**	${\mathrm{Int}}_{E_{0\to 0}}$
***c1***	0.03	0.05	3.36	0.001	3.27	3.39 × 10^−3^
***c2***	0.89	0.31	3.08	1.13	3.01	1.38
***c3***	0.99	0.28	3.11	1.43	3.00	2.44

## Data Availability

The original contributions presented in this study are included in the article/Supplementary Material. Further inquiries can be directed to the corresponding authors.

## Supplementary Materials

The following Supplementary Materials can be downloaded at: https://www.mdpi.com/article/10.3390/molecules30010044/s1, Figure S1: Comparisons of different models of FC|AH, FC|ASF, and FC|AS. The theoretical spectra have been redshifted by 0.35 eV for FC|AH, 0.35 eV for FC|ASF, and 0.43 eV for FC|AS.; Figure S2: Comparisons of different models of FC|VG, FC|VH, FC|AH, and FC|AS. The theoretical spectra have been redshifted by 0.42 for FC|VG, 0.39 eV for FC|VH, 0.35 eV for FC|AH, and 0.43 eV for FC|AS.; Figure S3: Comparisons of different models of FC|AH and FCHT|AH. The theoretical spectra have been redshifted by 0.35 eV.; Figure S4: Molecular orbital (MO), MO energies, and MO gaps (*E_g_*) of the ground state for the three [7]helicene derivatives.; Figure S5: Molecular orbital (MO), MO energies, and MO gaps (*E_g_*) of the relaxed S_1_ to the GS under S_1_-minima for the three [7]helicene derivatives.; Table S1: The intrafragment electron redistribution of fragmented and transferred electrons between fragments. (The data in the table refer to the number of electrons transferred.); Table S2: The frequencies (ω, cm^−1^) of the selected vibrational modes relevant for the vibronic structures of the spectra in FC|AH.; Table S3: The displacements in dimensionless units of the selected vibrational modes relevant for the vibronic structures of the spectra.; Table S4: Vertical excitation energies $E_{\mathrm{gf}}$ (eV), oscillator strengths ($\delta_{OPA}$), and rotatory strengths in length ($R_{length}$ in cgs) for the three [7]helicene derivatives.
