# Supplementary material for: Vibronic Effects Analysis of the Substituent Effect on the Spectral Properties of the EMI and CPL of Three [7]Helicene Derivatives
Source: Molecules. 2024 Dec 26;30(1):44. doi: 10.3390/molecules30010044 (PMC11721410; doi:10.3390/molecules30010044)
Supplement: Supplementary file 1 [file molecules-30-00044-s001.zip › molecules-3349290-supplementary.pdf]

# Vibronic Effects Analysis of the Substituent Effect on the Spectral Properties of the EMI and CPL of Three [7]Helicene Derivatives

**Qiushuang Xu[a,*], Meishan Wang[b, *], Yanli Liu[c]**

[a] *Department of Physics, Yantai University, Yantai 264005, China*

[b] *College of Integrated Circuits, Ludong University, 264025 Yantai, PR China*

[c] *School of Physics and Optoelectronics Engineering, Ludong University, 264025 Yantai, PR China*

* Corresponding authors: qsxu@ytu.edu.cn; mswang1971@163.com

## Table of Contents

1. Theoretical background of the spectrum lineshape ................................................. 3

2. IFCT calculations ........................................................................................ 4
3. Frequencies of selected vibrational modes ................................................ 4
3. Displacements of selected vibrational modes ............................................ 5
4. Comparisons of different models ............................................................... 5
5. Transition nature of ground state ................................................................ 7
6. Transition nature of the first excited state.................................................. 9

## 1. Theoretical background of the spectrum lineshape

In a time-independent (TI) formalism, the general lineshape of EMI and CPL can be expressed as:

$$L_{EMI}(\omega)=\sum_{f}\sum_{v_i,v_f} Z_i^{-1}e^{-\beta\hbar\omega_{vi}}g(\omega,\omega_{iv_i f v_f})\times\sum_{\alpha=x,y,z}\langle iv_i|\mu^\alpha|fv_f\rangle\langle fv_f|\hat{\mu}^\alpha|iv_i\rangle \quad (1)$$

$$L_{CPL}(\omega)=\sum_{f}\sum_{v_i,v_f} Z_i^{-1}e^{-\beta\hbar\omega_{vi}}g(\omega,\omega_{iv_i f v_f})\times\sum_{\alpha=x,y,z}\mathrm{Im}\langle iv_i|\mu^\alpha|fv_f\rangle\langle fv_f|\hat{m}^\alpha|iv_i\rangle$$

where $\beta=(k_BT)^{-1}$, $k_B$ is the Boltzmann constant and T is temperature. Moreover, $Z_i^{-1}\exp(-\hbar\omega_{iv_i}/k_BT)$ is the thermal population of $|v_i\rangle$ (the initial vibrational state), and $Z_i$ is the vibrational partition function of the initial sate. $g(\omega,\omega_{iv_i fv_f})$ is a broadening function, which is chosen to be a Gaussian function in the present work. $\omega_{iv_i fv_f}=(\omega_{fv_f}-\omega_{iv_i}),\omega$ is the circular frequency of the incident/emitted photon.

$\hat{\mu}$ and $\hat{m}$ are the electric and magnetic dipole moment operators, respectively, which can be explicitly evaluated as:

$$\mu^\alpha_{ivifv_f}=\langle v_i|\langle i|\hat{\mu}^\alpha|f\rangle|v_f\rangle=\langle v_i|\mu^\alpha_{e,if}(\boldsymbol{Q}_\kappa)|v_f\rangle \quad (2a)$$

$$m^\alpha_{ivifv_f}=Im\langle v_i|\langle i|\hat{m}^\alpha|f\rangle|v_f\rangle=\langle v_i|m^\alpha_{e,if}(\boldsymbol{Q}_\kappa)|v_f\rangle \quad (2b)$$

Here, the vector $\boldsymbol{Q}_\kappa$ is the set of normal coordinates $\{Q_a\}$ of either the initial or the final electronic state ($\kappa=i,f$). $\mu^\alpha_{e,if}(\boldsymbol{Q}_\kappa)$ and $m^\alpha_{e,if}(\mathbf{Q}_\kappa)$ are the so-called electronic transition dipole moments.

To obtain fully converged spectra, eqs (1) is transformed to a TD expression:

$$L_\chi(\omega)=\frac{1}{2\pi Z_i}\int_{-\infty}^{\infty}dt\left[\chi_{FC}(t,T)+\chi_{FC/HT}(t,T)+\chi_{HT/HT}(t,T)\right]e^{\pm i\omega t-\frac{\Gamma^2}{2}t^2} \quad (3)$$

$\chi$ represents EMI, and CPL, respectively. The "$\pm$" sign depends on the type of transition; the positive sign is for EMI and CPL. The quantities $\chi_{FC}(t,T)$, $\chi_{FC/HT}(t,T)$, and $\chi_{HT/HT}(t,T)$ in eqs. (3) are correlation functions whose expressions are analytical for harmonic PESs [1].

## 2. IFCT calculations

**Table S1** The intrafragment electron redistribution of fragmented and transferred electrons between fragments. (The data in the table refer to the number of electrons transferred.)

| molecules | A | B | C | A→B | A→C | B→C |
|---|---|---|---|---|---|---|
| ***c1*** | 0.998 | 0.00 | 0.00 | 0.00039 | 0.00017 | 0.00 |
| ***c2*** | 0.916 | 0.00062 | 0.00 | 0.069 | 0.00016 | 0.00 |
| ***c3*** | 0.900 | 0.00071 | 0.00005 | 0.066 | -0.00377 | -0.00071 |

## 3. Frequencies of selected vibrational modes

**Table S2** The frequencies ($\omega$, $cm^{-1}$) of the selected vibrational modes relevant for the vibronic structures of the spectra in FC|AH.

| | ***c1*** | ***c2*** | ***c3*** |
|---|---|---|---|
| 1 | 34.55 | 38.48 | 23.94 |
| 2 | 52.6 | 42.7 | 38.48 |
| 3 | 55.14 | 51.52 | 38.59 |
| 4 | 87.55 | 67.47 | 50.5 |
| 5 | 96.27 | 76.12 | 60.38 |
| 11 | 226.42 | 166.89 | 120.11 |
| 12 | 264.79 | 183.9 | 135.18 |
| 19 | 413.18 | 333.37 | 219.28 |
| 25 | 490.95 | 433.98 | 318.88 |
| 97 | 1397.17 | 1298.91 | 1183.65 |
| 99 | 1404.84 | 1341.23 | 1196.96 |
| 104 | 1472.61 | 1403.05 | 1229.67 |
| 105 | 1485.51 | 1404.05 | 1231.06 |
| 106 | 1497.4 | 1412.37 | 1233.15 |
| 117 | 1685.92 | 1570.64 | 1400.36 |
| 118 | 1692.18 | 1588.87 | 1400.93 |

## 3. Displacements of selected vibrational modes

**Table S3** The displacements in dimensionless units of the selected vibrational modes relevant for the vibronic structures of the spectra.

| | | | | | | | | |
|---|---|---|---|---|---|---|---|---|
| ***c1*** | $S_0$ mode | 1 | 2 | 11 | 12 | 97 | 99 | |
| | δ | 0.49 | 1.00 | 0.53 | -0.54 | 0.79 | 0.76 | |
| ***c2*** | $S_0$ mode | 3 | 4 | 16 | 19 | 104 | | |
| | δ | 1.28 | 0.61 | 0.62 | -0.71 | 0.96 | | |
| ***c2\|AS*** | $S_0$ mode | 3 | 4 | 19 | 105 | 106 | | |
| | δ | -0.94 | 0.90 | 0.76 | 0.79 | 0.53 | | |
| ***c3*** | $S_0$ mode | 1 | 2 | 5 | 23 | 25 | 117 | 118 |
| | δ | 0.51 | 0.89 | 0.66 | 0.44 | -0.78 | 0.62 | -0.76 |

## 4. Comparisons of different models

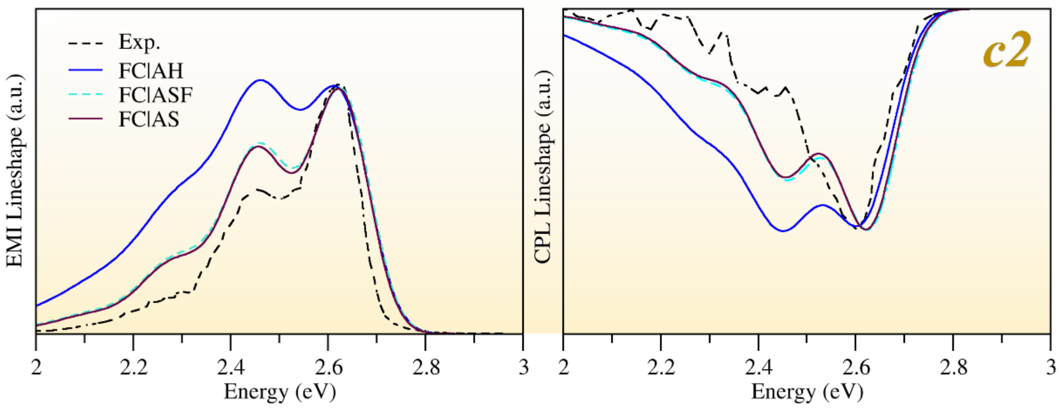


**Figure S1** Comparisons of different models of FC|AH, FC|ASF, and FC|AS. The theoretical spectra have been redshifted by 0.35 eV for FC|AH, 0.35 eV for FC|ASF, and 0.43 eV for FC|AS.

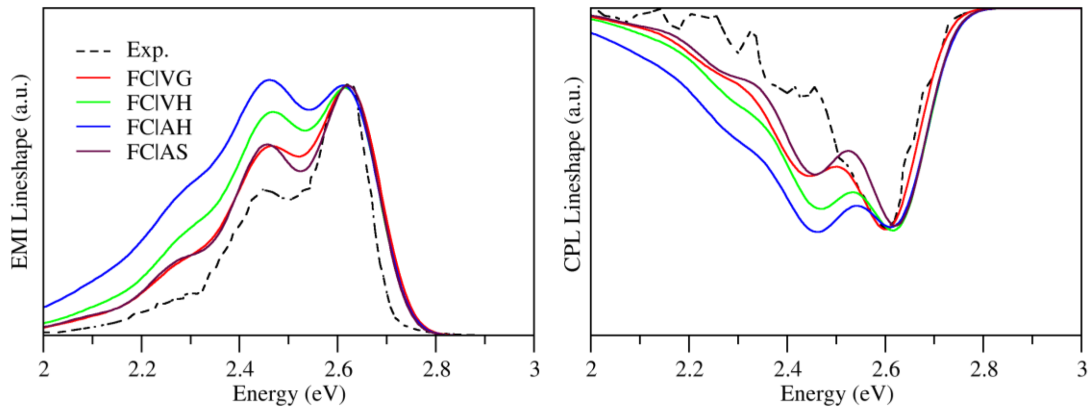


**Figure S2** Comparisons of different models of FC|VG, FC|VH, FC|AH, and FC|AS. The theoretical spectra have been redshifted by 0.42 for FC|VG, 0.39 eV for FC|VH, 0.35 eV for FC|AH, and 0.43 eV for FC|AS.

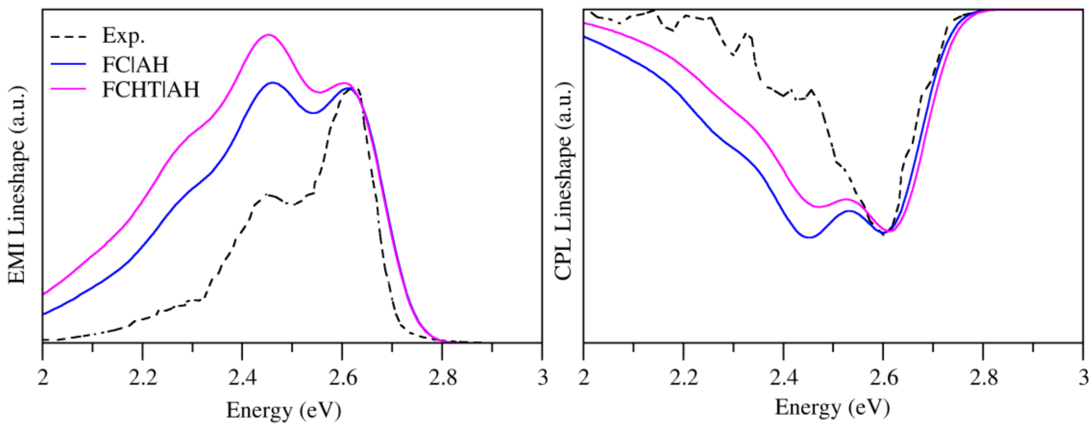


**Figure S3** Comparisons of different models of FC|AH and FCHT|AH. The theoretical spectra have been redshifted by 0.35 eV.

## 5. Transition nature of ground state

Table S4 displays some crucial parameters for the first three excited states of the three [7]helicene derivatives, vertical excitation energies $E_{gf}$ (eV), oscillator strengths ($\delta_{\mathrm{OPA}}$), rotatory strengths in length ($R_{length}$ in cgs), the nature of the transition, and the corresponding coefficient. We can see from Table S4 that the third ($S_3$) excited state is the strong state of ***c1***. The $S_3$ transition of ***c1*** arises from contributions associated with the HOMO-1-to-LUMO transitions. The second ($S_2$) excited state is the strong state of ***c2*** and ***c3***, originating from HOMO-1→LUMO and HOMO→LUMO, respectively. To illuminate the substituent effect, we show the corresponding molecular orbital (MO), MO energies, and MO gaps ($E_g$) of these three strong states in Figure S4. From the Figure S4, we can clearly see that the MO gaps decrease with the introduction of cyano groups and methoxy groups, and after the introduction of the methoxy groups do not change much compared with the cyano groups. This proves that, for the absorption process, the spectrum will be blueshifted, and the energy difference between the highest peaks of ***c2*** and ***c3*** will be almost the same.

**Table S4** Vertical excitation energies $E_{gf}$ (eV), oscillator strengths ($\delta_{\mathrm{OPA}}$), and rotatory strengths in length ($R_{length}$ in cgs) for the three [7]helicene derivatives.

| ***c1*** | | | | | |
|---|---|---|---|---|---|
| State | $E_{gf}$ | $\delta_{OPA}$ | $R_{length}$ | Transition | Coefficient |
| $S_1$ | 3.53 | 0.00 | -0.18 | HOMO-1→LUMO+1 | 0.44 |
| | | | | HOMO→LUMO | 0.50 |
| $S_2$ | 3.73 | 0.03 | 213.67 | HOMO-1→LUMO | 0.64 |
| $S_3$ | 3.83 | 0.25 | 890.22 | HOMO→LUMO+1 | 0.63 |
| ***c2*** | | | | | |
| State | $E_{gf}$ | $\delta_{OPA}$ | $R_{length}$ | Transition | Coefficient |
| $S_1$ | 3.27 | 0.05 | -57.02 | HOMO→LUMO | 0.63 |
| $S_2$ | 3.45 | 0.18 | 367.40 | HOMO-1→LUMO | 0.69 |

| State | $E_{gf}$ | $\delta_{OPA}$ | $R_{length}$ | Transition | Coefficient |
|---|---|---|---|---|---|
| $S_3$ | 3.70 | 0.04 | 197.11 | HOMO-2→LUMO | 0.51 |
| ***c3*** | | | | | |
| State | $E_{gf}$ | $\delta_{OPA}$ | $R_{length}$ | Transition | Coefficient |
| $S_1$ | 3.28 | 0.07 | -61.16 | HOMO-1→LUMO | 0.57 |
| $S_2$ | 3.33 | 0.18 | 423.42 | HOMO→LUMO | 0.68 |
| $S_3$ | 3.72 | 0.03 | 253.48 | HOMO-1→LUMO+1 | 0.50 |
| | | | | HOMO-3→LUMO | -0.42 |

HOMO: The highest occupied molecular orbital.

LUMO: The lowest unoccupied molecular orbital transitions.

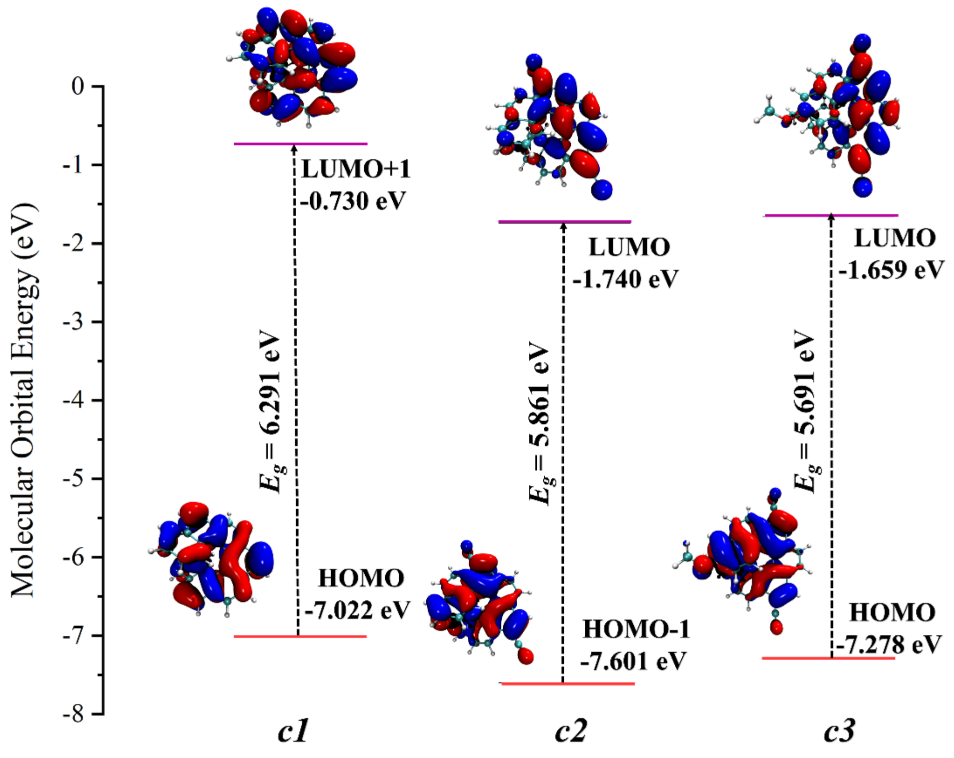


**Figure S4** The molecular orbital (MO), MO energies, and MO gaps ($E_g$) of the ground state for the three [7]helicene derivatives.

## 6. The transition nature of the first excited state

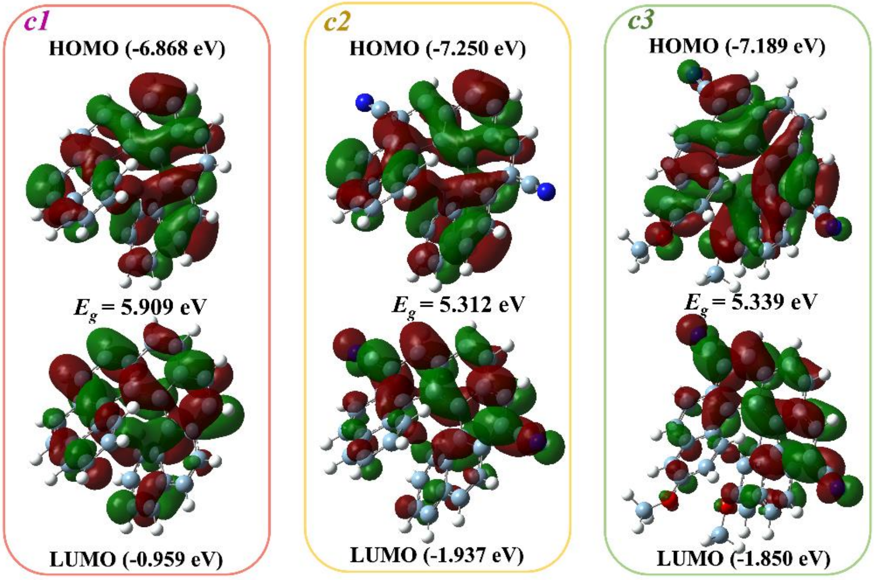


**Figure S5** The molecular orbital (MO), MO energies, and MO gaps ($E_g$) of the relaxed $S_1$ to the GS under $S_1$-minima for the three [7]helicene derivatives.

References


1. Avila Ferrer, F. J.; Cerezo, J.; Soto, J.; Improta, R.; Santoro, F., First-principle computation of absorption and fluorescence spectra in solution accounting for vibronic structure, temperature effects and solvent inhomogenous broadening. *Comput. Theo. Chem.* **2014,** 1040, 328-337.
